# Recapture or Precapture? Fallibility of Standard Capture-Recapture Methods in the Presence of Referrals Between Sources

**DOI:** 10.1093/aje/kwu056

**Published:** 2014-04-11

**Authors:** Hayley E. Jones, Matthew Hickman, Nicky J. Welton, Daniela De Angelis, Ross J. Harris, A. E. Ades

**Keywords:** bias, log-linear models, model selection, parameter identifiability, problem drug use, prevalence estimation

## Abstract

Capture-recapture methods, largely developed in ecology, are now commonly used in epidemiology to adjust for incomplete registries and to estimate the size of difficult-to-reach populations such as problem drug users. Overlapping lists of individuals in the target population, taken from administrative data sources, are considered analogous to overlapping “captures” of animals. Log-linear models, incorporating interaction terms to account for dependencies between sources, are used to predict the number of unobserved individuals and, hence, the total population size. A standard assumption to ensure parameter identifiability is that the highest-order interaction term is 0. We demonstrate that, when individuals are referred directly between sources, this assumption will often be violated, and the standard modeling approach may lead to seriously biased estimates. We refer to such individuals as having been “precaptured,” rather than truly recaptured. Although sometimes an alternative identifiable log-linear model could accommodate the referral structure, this will not always be the case. Further, multiple plausible models may fit the data equally well but provide widely varying estimates of the population size. We demonstrate an alternative modeling approach, based on an interpretable parameterization and driven by careful consideration of the relationships between the sources, and we make recommendations for capture-recapture in practice.

Capture-recapture (CRC) methods are widely used in epidemiology to adjust for incomplete ascertainment by disease registries and to estimate the size of elusive populations (1–4). A CRC data set consists of overlapping lists of individuals in the target population taken from administrative data sources. This is considered akin to multiple captures and recaptures in animal abundance studies (5, 6). The observed overlaps are used to estimate the size of the unobserved population and, hence, the total population size. CRC is particularly useful for providing prevalence estimates and denominators for populations that are underestimated by standard sampling methods, for example, problem drug users (PDUs) (7–20), commercial sex workers, the homeless, and individuals with subclinical disease (21–25). In Europe, CRC is the recommended method for estimating the size of PDU populations (26) and, in the United Kingdom, CRC estimates are used to monitor the effectiveness of drug policy (27, 28).

Generally, log-linear regressions are used to model the observed data, assuming a multinomial or (equivalently) Poisson likelihood (29). Aspects requiring careful consideration include the possibility of dependencies between data sources, whereby the appearance of an individual in 1 source affects his or her probability of appearance in another, as well as heterogeneities in capture probabilities, such that specific subgroups of the population are more or less likely to be recorded. These 2 phenomena are closely related (2, 30), but in some cases the latter may be accounted for by model stratification or the incorporation of covariates in the regression model (31, 32). In this paper, we focus instead on issues relating to dependencies between data sources that cannot be accounted for by using measured covariates. An early suggestion to merge dependent data sources (33) has now been subsumed within the more general approach of incorporation of source-by-source interaction terms in a log-linear model (2, 29, 34).

We will demonstrate that this standard approach is often inadequate in the presence of referrals of individuals between sources, a mechanism we believe to be very common in CRC studies of human populations. When a proportion of individuals “captured” in 1 source is referred to another, these individuals can be thought of as having been “precaptured” rather than truly “recaptured.” Because they have simply been passed from 1 source to another, they cannot be informative about the population size. Whereas more general source dependencies in epidemiologic CRC have their counterpart in ecological CRC (broadly comparable to “trap fascination” or “trap avoidance” (4)), direct referrals would be analogous to catching a sample of fish in a net, setting some proportion of these fish aside, and letting them contribute to the second sample.

The critical issues are those of model selection and parameter identification. Often, referrals will not be the only mechanism creating between-source dependencies, so that the full dependency structure might be quite complex. In a CRC analysis of S data sources, inclusion of all possible interaction terms in a log-linear model would require 2^S^ parameters—1 more than the number of observations (29). It is therefore necessary to enforce some constraint, such as assuming that at least 1 of the possible interaction terms is 0. The usual approach is to consider only models with no S-way interaction (2). This is motivated by the view that higher-order interactions are unlikely in the absence of their lower-order relatives, sometimes referred to as the “hierarchy principle” (4, 29). However, Cormack (35) describes the no S-way interaction assumption as an “act of faith.” Variable catchability (heterogeneity) and migration are 2 phenomena that have been recognized to induce interactions of an order higher than 1 (1, 36).

Using the 3-source case as an example, we will show that between-source referrals will often correspond to a 3-way interaction term in the log-linear model. Sometimes an alternative saturated log-linear model would adequately accommodate the referral structure. However, alternative saturated models can lead to widely different estimates of the population size and, because all fit the data perfectly by definition, it is impossible to choose between them without expert knowledge of the interrelationships between the sources. Further, in slightly more complex scenarios, we will show that the true model structure cannot be represented by any identifiable log-linear model.

## MOTIVATING EXAMPLE: PREVALENCE OF PROBLEM DRUG USE IN ENGLAND

Hay et al. (27) estimated the number of PDUs (i.e., users of opiates and/or crack cocaine) aged 15–64 years in England in the 1-year period from April 2009 to March 2010. Lists of PDUs identified in each of the following 4 administrative data sources during that time period were obtained and linked: “treatment in the community,” “arrest for possession,” “probation,” and “prison.” For ease of exposition, we discard the prison source but return to discuss this later. We also aggregate over the available covariate values (age group, sex, and geographical area). For a description of the data sources and the process used for matching individuals, see the article by Hay et al. (37).

The aggregated data are shown in Table 1. We refer to the sources as S1, S2, and S3, and we denote the observed number of individuals in each cell as *x_ijk_*, where the indices indicate presence (subscript = 1) or absence (subscript = 0) in each of the 3 sources. For example, *x*_101_ denotes the number of individuals observed in S1 and S3 but not in S2. On occasion, we will write S*u* = 1 and S*u* = 0 to represent presence or absence in Source *u* (*u* = 1,2,3).

**Table 1.** Observed Numbers of Problem Drug Users, England, 2009–2010

Treatment (Source 1)^a^	Arrest (Source 2)^a^	Probation (Source 3)^a^	Notation for Observed No. of Individuals	Observed Data (Total = 192,551)
Yes	Yes	Yes	*x*_111_	1,868
Yes	Yes	No	*x*_110_	3,123
Yes	No	Yes	*x*_101_	21,633
Yes	No	No	*x*_100_	147,049
No	Yes	Yes	*x*_011_	349
No	Yes	No	*x*_010_	4,491
No	No	Yes	*x*_001_	14,038
No	No	No	*x*_000_	Unobserved

^a^ “Yes” denotes presence, and “no” denotes absence from the data source.

Clearly, we would expect some dependencies among these 3 data sources. In particular, we would anticipate a positive dependency between arrest and probation, because we would expect individuals committing 1 crime to be more likely to commit another. A negative dependency between treatment and the 2 criminal justice system sources is also possible, although these might be independent. The dependency structure is further complicated by the presence of referrals between sources. Specifically, individuals arrested for drug possession may be put on probation, and referrals to drug treatment are made from each of the criminal justice system sources.

Using the standard log-linear modeling framework, if we adopt the no S-way interaction rule, there are only 8 possible models to consider. These are indicated in Table 2, where we denote, for example, an S1 by S2 interaction by “S1xS2”. Model 1, which we refer to as the base case or simply “base,” assumes independence of the 3 sources. Alternatively, we can include 1 (models 2–4), 2 (models 5–7), or all 3 (model 8) source-by-source interaction terms. Model 8 is a saturated model, because it involves fitting 7 parameters to the 7 observed data points. In addition, we present results from 3 alternative saturated log-linear models (models 9–11), which violate the no S-way interaction rule and therefore are not usually considered in CRC applications. Each involves excluding 1 of the 2-way interaction terms in favor of accommodating a 3-way interaction.

**Table 2.** Results From Poisson Log-Linear Regression Analyses of the Problem Drug User Data, England, 2009–2010

Log-Linear Model		Estimated Missing Cell Count^a^		Estimated Total Population Size^a,b^		Model Fit^c^		
Model No.	Description	*◯*_000_	95% CI	*N̂*	95% CI	AIC	*G*^2^	df
1	Base	100,000	98,200, 102,000	292,600	290,700, 294,500	2,501	2,419	3
2	Base + S1xS2	91,000	89,100, 93,000	283,600	281,700, 285,600	2,128	2,043	2
3	Base + S1xS3	153,700	147,700, 159,900	346,200	340,200, 352,500	1,948	1,864	2
4	Base + S2xS3	104,300	102,300, 106,300	296,800	294,800, 298,900	1,779	1,694	2
5	Base + S1xS2 + S1xS3	180,600	161,800, 201,700	373,200	354,400, 394,200	1,940	1,854	1
6	Base + S1xS2 + S2xS3	95,400	93,400, 97,500	288,000	285,900, 290,100	1,474	1,388	1
7	Base + S1xS3 + S2xS3	211,500	202,000, 221,400	404,000	394,500, 414,000	645	558	1
8	Base + S1xS2 + S1xS3 + S2xS3	734,500	648,200, 832,300	927,000	840,700, 1,024,800	88	0	0
9	Base + S1xS2 + S1xS3 + S1xS2xS3	180,600	161,800, 201,700	373,200	354,400, 394,200	88	0	0
10	Base + S1xS2 + S2xS3 + S1xS2xS3	95,400	93,400, 97,500	288,000	285,900, 290,100	88	0	0
11	Base + S1xS3 + S2xS3 + S1xS2xS3	211,500	202,000, 221,400	404,000	394,500; 414,000	88	0	0

Abbreviations: AIC, Akaike information criterion; CI, confidence interval.

^a^ All population size estimates have been rounded to the nearest 100.

^b^ This is simply the estimated missing cell count plus the observed number of 192,551 (Table 1).

^c^ *G*^2^ = the likelihood ratio test statistic, displayed with the degrees of freedom for the corresponding χ^2^ test (*P* values are omitted because, for all nonsaturated models, *P* < 0.001).

As saturated models, models 8–11 will all fit the data perfectly, but they will provide different extrapolations to the missing cell. It can be shown that the maximum likelihood estimators for the missing cell based on models 9–11 are in fact identical to those from models 5–7. For example, models 5 and 9 both estimate *x*_000_ by *x*_010_*x*_001_/*x*_011._ This arises from both models assuming independence between S2 and S3 in the S1 = 0 cells. In estimating *x*_000_, all 4 S1 = 1 cells are ignored. However, model 9, unlike model 5, does not enforce the assumption of independence between S2 and S3 among the S1 = 1 cells, and hence will result in better model fit (at the cost of an additional parameter).

Model selection in epidemiologic CRC is often carried out by minimizing some information criterion such as the Akaike information criterion (AIC) (1, 29). Alternatively, the simplest model is selected that fits the data according to a likelihood ratio test statistic *G*^2^, referred to a χ^2^ distribution (1, 29). In Table 2 we show the AIC and *G*^2^ statistics for each of the 11 models, in addition to estimates of the population size. Estimates from models incorporating the available covariates were similar (not shown). We adopt the most common definition of the AIC (38, 39), but note that an alternative version is popular in the CRC literature. The 2 definitions differ only in terms of a constant; because AICs should be interpreted only relative to each other rather than in absolute terms, the distinction is not important. Regression models were fitted using Stata, version 13, statistical software (StataCorp LP, College Station, Texas), assuming a Poisson likelihood. We coded all models such that the presence or absence in a source was indicated by 1 or 0, respectively. Other parameterizations can be used, but care must be taken to interpret the interaction models accordingly.

Table 2 shows that the 4 saturated models jointly have by far the lowest AIC. The *G*^2^ statistic is seen to be very large relative to its degrees of freedom for all other models, indicating that none of these provides an adequate fit to the data. The 4 saturated models are seen to imply widely different estimates of the total population size *N*, ranging from 288,000 to 927,000 and with 95% confidence intervals not overlapping.

Following the no S-way interaction rule, models 9–11 would not be considered, so model 8 would be selected. However, this is seen to provide an estimate of 927,000 (95% confidence interval (CI): 840,700, 1,024,800) PDUs in England, which is more than 3 times the magnitude of the previously published estimate (27) and would imply that 2.7% of adults aged 15–64 years were heroin or crack cocaine users. This is unsupported by other evidence (such as surveys of the proportion of people in treatment and the number of drug-related deaths) and is considered infeasible by experts. Perhaps the prevalence estimate from 1 of the other saturated models is more accurate, but there is no obvious reason to select 1 of these over the others. Moreover, we will demonstrate below that it is perfectly plausible that none of these 4 saturated models provides an accurate estimate of the population size, even in the absence of realistic additional complications, such as variable catchability or errors made in the matching process.

If the “prison” source is also included then, following the hierarchy principle, there are 113 possible log-linear models (2). The best fitting of these was found to be the saturated model with six 2-way and four 3-way interactions between sources. This model gave a still more implausible estimate of 1,614,000 (95% CI: 1,167,900, 2,265,000) PDUs in England. Again, alternative saturated models provided very different estimates. The methodological problems are therefore clearly not confined to 3-source models.

## BIAS DUE TO BETWEEN-SOURCE REFERRAL

To assess whether models incorporating referrals between sources can be fitted within the standard log-linear framework, we compare the form of the expected cell counts under various scenarios, focusing again on the 3-source case for simplicity. We denote the conditional probability of an individual appearing in source *u* given presence or absence in source *v*, before any referrals take place, by *p_u_*_|S*v* = 1_ and *p_u_*_|S*v* = 0_, respectively. When *p_u_*_|S*v* = 1_ = *p_u_*_|S*v* = 0_, we simply write *p_u_*.

We consider 3 simple referral scenarios as examples. The expected cell counts under each of these, before and after referrals, are shown in Table 3. In the first 2 scenarios, there are assumed to be no “standard” interactions (i.e., if it were not for the referrals, then the data sources would be independent). In referral scenario 1, a proportion *q*_13_ of individuals in S1 are referred to S3. This occurs independently of whether they were seen in S2 or would otherwise have been seen in S3 anyway. From the postreferral expected cell counts, it can be seen that this is equivalent to a standard interaction between S1 and S3, with *p*_3|S1=1_ = *p*_3_ + *q*_13_(1 − *p*_3_) and *p*_3|S1=0_ = *p*_3_. Hence, this very simple scenario corresponds to model 3 in Table 2.

**Table 3.** Expected Cell Counts in a 3-Source Capture-Recapture Under Hypothetical Referral Scenarios

Source Combination^a^			Expected Cell Counts	
S1	S2	S3	Prereferral	Postreferral
*Referral Scenario 1*^b^				
1	1	1	*Np*_1_*p*_2_*p*_3_	*Np*_1_*p*_2_[*p*_3_ + *q*_13_(1 − *p*_3_)]
1	1	0	*Np*_1_*p*_2_(1 − *p*_3_)	*Np*_1_*p*_2_{1 − [*p*_3_ + *q*_13_(1 − *p*_3_)]}
1	0	1	*Np*_1_(1 − *p*_2_)*p*_3_	*Np*_1_(1 − *p*_2_)[*p*_3_ + *q*_13_(1 − *p*_3_)]
1	0	0	*Np*_1_(1 − *p*_2_)(1 − *p*_3_)	*Np*_1_(1 − *p*_2_){1 − [*p*_3_ + *q*_13_(1 − *p*_3_)]}
0	1	1	*N*(1 − *p*_1_)*p*_2_*p*_3_	*N*(1 − *p*_1_)*p*_2_*p*_3_
0	1	0	*N*(1 − *p*_1_)*p*_2_(1 − *p*_3_)	*N*(1 − *p*_1_)*p*_2_(1 − *p*_3_)
0	0	1	*N*(1 − *p*_1_)(1 − *p*_2_)*p*_3_	*N*(1 − *p*_1_)(1 − *p*_2_)*p*_3_
0	0	0	*N*(1 − *p*_1_)(1 − *p*_2_)(1 − *p*_3_)	*N*(1 − *p*_1_)(1 − *p*_2_)(1 − *p*_3_)
*Referral Scenario 2*^c^				
1	1	1	*Np*_1_*p*_2_*p*_3_	*Np*_1_*p*_2_[*p*_3_ + (*q*_13_ + *q*_23_ − *q*_13_*q*_23_) (1 − *p*_3_)]
1	1	0	*Np*_1_*p*_2_(1 − *p*_3_)	*Np*_1_*p*_2_{1 − [*p*_3_ + (*q*_13_ + *q*_23_ − *q*_13_*q*_23_)(1 − *p*_3_)]}
1	0	1	*Np*_1_(1 − *p*_2_)*p*_3_	*Np*_1_(1 − *p*_2_)[*p*_3_ + *q*_13_(1 − *p*_3_)]
1	0	0	*Np*_1_(1 − *p*_2_)(1 − *p*_3_)	*Np*_1_(1 − *p*_2_){1 − [*p*_3_ + *q*_13_(1 − *p*_3_)]}
0	1	1	*N*(1 − *p*_1_)*p*_2_*p*_3_	*N*(1 − *p*_1_)*p*_2_[*p*_3_ + *q*_23_(1 − *p*_3_)]
0	1	0	*N*(1 − *p*_1_)*p*_2_(1 − *p*_3_)	*N*(1 − *p*_1_)*p*_2_{1 − [*p*_3_ + *q*_23_(1 − *p*_3_)]}
0	0	1	*N*(1 − *p*_1_)(1 − *p*_2_)*p*_3_	*N*(1 − *p*_1_)(1 − *p*_2_)*p*_3_
0	0	0	*N*(1 − *p*_1_)(1 − *p*_2_)(1 − *p*_3_)	*N*(1 − *p*_1_)(1 − *p*_2_)(1 − *p*_3_)
*Referral Scenario 3*^d^				
1	1	1	*Np*_1_*p*_2|S1=1_*p*_3|S2=1_	*N*[*p*_1_*p*_2|S1=1_ + *q*_31_(1 − *p*_1_)*p*_2|S1=0_]*p*_3|S2=1_
1	1	0	*Np*_1_*p*_2|S1=1_(1 − *p*_3|S2=1_)	*Np*_1_*p*_2|S1=1_(1 − *p*_3|S2=1_)
1	0	1	*Np*_1_(1 − *p*_2|S1=1_)*p*_3|S2=0_	*N*[*p*_1_(1 − *p*_2|S1=1_) + *q*_31_(1 − *p*_1_)(1 − *p*_2|S1=0_)]*p*_3|S2=0_
1	0	0	*Np*_1_(1 − *p*_2|S1=1_)(1 − *p*_3|S2=0_)	*Np*_1_(1 − *p*_2|S1=1_)(1 − *p*_3|S2=0_)
0	1	1	*N*(1 − *p*_1_)*p*_2|S1=0_*p*_3|S2=1_	*N*(1 − *p*_1_)(1 − *q*_31_)*p*_2|S1=0_*p*_3|S2=1_
0	1	0	*N*(1 − *p*_1_)*p*_2|S1=0_(1 − *p*_3|S2=1_)	*N*(1 − *p*_1_)*p*_2|S1=0_(1 − *p*_3|S2=1_)
0	0	1	*N*(1 − *p*_1_)(1 − *p*_2|S1=0_)*p*_3|S2=0_	*N*(1 − *p*_1_)(1 − *q*_31_)(1 − *p*_2|S1=0_)*p*_3|S2=0_
0	0	0	*N*(1 − *p*_1_)(1 − *p*_2|S1=0_)(1 − *p*_3|S2=0_)	*N*(1 − *p*_1_)(1 − *p*_2|S1=0_)(1 − *p*_3|S2=0_)

^a^ For example, the row with S1 = 1, S2 = 1, and S3 = 0 corresponds to the expected number of individuals observed in source 1 and source 2 but not in source 3 (the expectation of *x*_110_).

^b^ No standard interactions, but referral of a proportion *q*_13_ of individuals from source 1 into source 3. (Prereferral: base. Postreferral: base + referrals from S1 into S3).

^c^ No standard interactions, but referral of a proportion *q*_13_ of individuals from source 1 into source 3 and, independently, a proportion *q*_23_ of individuals from source 2 into source 3. (Prereferral: base. Postreferral: base + referrals from S1 into S3 + referrals from S2 into S3).

^d^ Source 1 by source 2 and source 2 by source 3 interactions, plus referral of a proportion *q*_13_ of individuals from source 1 into source 3. (Prereferral: base + S1xS2 + S2xS3. Postreferral: base + S1xS2 + S2xS3 + referrals from S1 into S3).

However if, in addition, a proportion *q*_23_ of individuals were independently referred from S2 into S3, then the expected cell counts would be as shown in the second part of Table 3 (referral scenario 2). As we would expect, the probability of an individual appearing in S3 now depends on appearance or otherwise in each of S1 and S2. However, we show in Appendix 1 that the corresponding log-linear model is not model 7 (base + S1xS3 + S2xS3) but actually model 11 (base + S1xS3 + S2xS3 + S1xS2xS3), violating the hierarchy principle. Specifically, this means that the referrals have induced a relationship between sources 1 and 2 among individuals observed in source 3, but not among individuals not seen in source 3. Clearly, even a simple scenario involving referrals can correspond to a 3-way interaction term in the log-linear model, so that none of the 8 standard models is appropriate.

In the final section of Table 3, we consider the scenario of 2 standard interactions (S1xS2 and S2xS3) followed by referral of a proportion *q*_31_ of individuals from S3 into S1. We show in Appendix 1 that the corresponding log-linear model requires both a 3-way interaction term and also an S1xS3 interaction term. With only 7 observed data points, it is not possible to fit the appropriate log-linear model, which would need to include 4 interaction terms, and therefore 8 parameters in total.

In summary, in a 3-source CRC analysis, some simple hypothetical referral scenarios can be parameterized as log-linear models with only 2-way interaction terms, whereas others induce a 3-way interaction. Sometimes, for example in our referral scenario 3, it will not be possible to accommodate the correct data structure using any identifiable log-linear model.

## ARTIFICIAL 3-SOURCE DATA SETS

We now consider 2 artificial data sets in order to demonstrate that standard methods can be very misleading. The 2 data sets, displayed in Table 4, were simulated from multinomial models under referral scenarios 2 and 3. Refer to the table's legend for full details. In both cases, we treat the cell count *x*_000_ as missing and estimate it using each of the 11 log-linear models. Population size estimates and model fit statistics are shown in Tables 5 and 6.

**Table 4.** Two Artificial Data Sets, Simulated From Multinomial Models With Referrals

Source Combinations^a^			Artificial Data Set 1^b^	Artificial Data Set 2^c^
S1	S2	S3		
1	1	1	4,397	8,155
1	1	0	3,637	1,616
1	0	1	25,613	18,209
1	0	0	46,312	61,087
0	1	1	5,226	22,964
0	1	0	6,714	19,430
0	0	1	21,506	11,425
0	0	0	86,595	57,114
Total observed			113,405	142,886
Total population size			200,000	200,000

^a^ For example, the row with S1 = 1, S2 = 1, and S3 = 0 shows *x*_110_, the number of individuals observed in source 1 and source 2 but not in source 3.

^b^ Simulated from referral scenario 2 in Table 3 (no standard interactions, but referral of a proportion *q*_13_ of individuals from source 1 into source 3 and, independently, a proportion *q*_23_ of individuals from source 2 into source 3), assuming the following parameter values: *N* = 200,000, *p*_1_ = 0.4, *p*_2_ = 0.1, *p*_3_ = 0.2, *q*_13_ = 0.2, and *q*_23_ = 0.3 (*N* = total population size, and *p_u_* = prereferral probability of appearing in source *u*).

^c^ Simulated from referral scenario 3 in Table 3 (standard source 1 by source 2 and source 2 by source 3 interactions, plus referral of a proportion *q*_13_ of individuals from source 1 into source 3), assuming the following parameter values: *N* = 200,000, *p*_1_ = 0.4, *p*_2|S1=0_ = 0.4, *p*_2|S1=1_ = 0.05, *p*_3|S2=0_ = 0.2, *p*_3|S2=1_ = 0.6, and *q*_13_ = 0.2 (*N* = total population size, and *p_u_*_|Sv=1_ and *p_u_*_|Sv=0_ are the prereferral probabilities of appearing in source *u*, given presence or absence in source *v*).

**Table 5.** Results From Poisson Log-Linear Regression Analyses Applied to Artificial Data Set 1

Log-Linear Model		Estimated Missing Cell Count^a,b^		Estimated Total Population Size^a,c^		Model Fit^d^		
Model No.	Description	*◯*_000_	95% CI	*N̂*	95% CI	AIC	*G*^2^	df
1	Base	41,000	40,300, 41,700	154,400	153,700, 155,100	2,825	2,739	3
2	Base + S1xS2	34,600	33,900, 35,200	148,000	147,300, 148,600	1,345	1,258	2
3	Base + S1xS3	47,300	45,900, 48,800	160,700	159,300, 162,200	2,705	2,617	2
4	Base + S2xS3	46,000	45,200, 46,900	159,400	158,600, 160,300	1,446	1,358	2
5	Base + S1xS2 + S1xS3	27,600	26,600, 28,700	141,000	140,000, 142,100	1,179	1,089	1
6	Base + S1xS2 + S2xS3	38,900	38,100, 39,700	152,300	151,500, 153,100	321	231	1
7	Base + S1xS3 + S2xS3	85,500	82,000, 89,100	198,900	195,400, 202,500	330	241	1
8	Base + S1xS2 + S1xS3 + S2xS3	60,400	56,900, 64,200	173,800	170,300, 177,600	92	0	0
9	Base + S1xS2 + S1xS3 + S1xS2xS3	27,600	26,600, 28,700	141,000	140,000, 142,100	92	0	0
10	Base + S1xS2 + S2xS3 + S1xS2xS3	38,900	38,100, 39,700	152,300	151,500, 153,100	92	0	0
11	Base + S1xS3 + S2xS3 + S1xS2xS3	85,500	82,000, 89,100	198,900	195,400, 202,500	92	0	0

Abbreviations: AIC, Akaike information criterion; CI, confidence interval.

^a^ All population size estimates have been rounded to the nearest 100.

^b^ Estimates of the missing cell count should be compared with the true value of 86,595 (Table 4).

^c^ This is simply the estimated missing cell count plus the observed number of 113,405 (Table 4). Estimates should be compared with the true value of 200,000.

^d^ *G*^2^ = the likelihood ratio test statistic, displayed with the degrees of freedom for the corresponding χ^2^ test (*P* values are omitted because, for all nonsaturated models, *P* < 0.001).

**Table 6.** Results From Poisson Log-Linear Regression Analyses Applied to Artificial Data Set 2

Log-Linear Model		Estimated Missing Cell Count^a,b^		Estimated Total Population Size^a,c^		Model Fit^d^		
Model No.	Description	*x̂*_000_	95% CI	*N̂*	95% CI	AIC	*G*^2^	df
1	Base	57,000	56,200, 57,800	199,900	199,100, 200,700	51,619	51,532	3
2	Base + S1xS2	19,000	18,600, 19,400	161,900	161,500, 162,300	21,365	21,276	2
3	Base + S1xS3	53,800	52,800, 54,900	196,700	195,700, 197,800	51,544	51,454	2
4	Base + S2xS3	117,500	115,600, 119,500	260,400	258,500, 262,300	18,469	18,380	2
5	Base + S1xS2 + S1xS3	9,700	9,400, 9,900	152,600	152,300, 152,800	14,078	13,986	1
6	Base + S1xS2 + S2xS3	38,300	37,400, 39,300	181,200	180,300, 182,200	3,210	3,119	1
7	Base + S1xS3 + S2xS3	734,500	697,700, 773,200	877,400	840,600, 916,100	7,936	7,845	1
8	Base + S1xS2 + S1xS3 + S2xS3	163,700	153,800, 174,100	306,500	296,700, 317,000	93	0	0
9	Base + S1xS2 + S1xS3 + S1xS2xS3	9,700	9,400, 9,900	152,600	152,300, 152,800	93	0	0
10	Base + S1xS2 + S2xS3 + S1xS2xS3	38,300	37,400, 39,300	181,200	180,300, 182,200	93	0	0
11	Base + S1xS3 + S2xS3 + S1xS2xS3	734,500	697,700, 773,200	877,400	840,600, 916,100	93	0	0

Abbreviations: AIC, Akaike information criterion; CI, confidence interval.

^a^ All population size estimates have been rounded to the nearest 100.

^b^ Estimates of the missing cell count should be compared with the true value of 57,114 (Table 4).

^c^ This is simply the estimated missing cell count plus the observed number of 142,886 (Table 4). Estimates should be compared with the true value of 200,000.

^d^ *G*^2^ = the likelihood ratio test statistic, displayed with the degrees of freedom for the corresponding χ^2^ test (*P* values are omitted because, for all nonsaturated models, *P* < 0.001).

As with our real data, we see that the 4 saturated models (models 8–11) jointly have by far the lowest AIC statistics. All other models have very large *G*^2^ statistics relative to their degrees of freedom, indicating inadequate fit to the data. Once again, of the saturated models, the default choice would be model 8 because of the no S-way interaction rule. For the first data set, the resulting estimate of the missing cell is 60,400 (95% CI: 56,900, 64,200), with the 95% confidence interval not incorporating the true value of 86,600 (Table 4). For the second data set, the estimate of the missing cell is 163,700 (95% CI: 153,800, 174,100), which is much greater than the true value (Table 4) of only 57,100. For each data set, the alternative saturated models generate widely differing estimates of the hidden population size.

For artificial data set 1, we know from the section “Bias due to between-source referral” that the appropriate log-linear model is model 11, and we see in Table 5 that this does indeed recover the true value accurately, estimating the missing cell count to be 85,500 (95% CI: 82,000, 89,100). However, without expert knowledge of the relationships among the 3 data sources and the mathematical analysis above, it would be impossible to choose among models 8–11 if all were considered. As noted above, model 7 also gives the correct maximum likelihood estimate. However, this model fits the observed data poorly and, as such, would not be selected by analysts in practice.

We showed in the section titled “Bias due to between-source referral” and in Appendix 1 that none of the 11 log-linear models corresponds to the truth for the model used to generate artificial data set 2. In fact, none of the 4 saturated models in Table 6 produces a 95% confidence interval that includes the true value. If, however, we knew the underlying structure of the data, then it would be possible to obtain an unbiased estimate by formulating a model directly in terms of meaningful parameters (probabilities and proportions) of the type in Table 3. As an example, in Appendix 2 we present WinBUGS (40) code for fitting the correct model to artificial data set 2. Here we assume uninformative uniform(0,1) prior distributions for each of the *p* and *q* parameters and a vague half-normal distribution for the log of the total population size. Fitting this model, the resulting estimate of the unseen population size was 56,900 (95% credible interval: 55,000, 58,800), implying a total population size of 199,800 (95% credible interval: 197,900, 201,700), which is extremely close to the true value of 200,000. Note that, because this “correct” model is 1 of many possible saturated models (others including models 8–11 in Table 6), we would not possibly know to select it without external knowledge of the data structure.

## DISCUSSION

We have demonstrated that, in the presence of referral mechanisms between sources, the standard log-linear modeling approach to CRC is too restrictive and can lead to grossly misleading prevalence estimates. The absolute bias will be largest when the proportion of the population observed is low. Model averaging (41) is not an appropriate solution if none of the models considered is “correct” and/or if only saturated models fit the data, because potentially widely varying estimates from different saturated models would be assigned equal weight.

As early as 1968, Wittes and Sidel (42) noted cause for concern if it is suspected that information from 1 CRC source has been obtained directly from another. This was before the advent of log-linear models. Inclusion of interaction terms in a log-linear model is now the standard methodology for accounting for source dependencies (2, 29), with the highest-order interaction term assumed to equal 0. We are not the first to point out that this approach is not infallible (35, 36). In particular, Hook and Regal (2) have suggested a need for caution when the standard saturated model is selected by some criterion such as the AIC. However, the fact that alternative saturated models can provide such dramatically different estimates of the population size does not appear to have been recognized. For example, Regal and Hook (36) demonstrated an application with likely violation of the “no S-way interaction” rule, but the inferred interaction was small, so that ignoring it was found to have little effect on the estimate of the population size.

We highlight precapture as a particular source of concern because we believe that many CRC studies in epidemiology are likely to involve referrals between sources and might therefore be subject to bias. Policies that seek to move drug users identified in the criminal justice system into treatment (43) complicate attempts to estimate the prevalence of drug addiction using CRC. More generally, applications of CRC in epidemiology frequently combine reports from clinical and laboratory sources, from primary care and secondary care settings, or from different medical specialists (44). It seems almost inevitable that some referrals will occur between such sources. For example, Van Hest et al. (45) used infectious disease notifications, laboratory reports, and data on hospitalizations to estimate tuberculosis prevalence, but they noted that “[tuberculosis] services in England are organized around close collaboration between clinicians, microbiologists and public health professionals.” Similarly, CRC has been used to estimate the incidence of congenital cataract by combining information from pediatricians and pediatric ophthalmologists (46). However, cataract is often a manifestation of a wider dysmorphic syndrome. Good practice dictates that children who present first to pediatricians will be referred to ophthalmologists, whereas those presenting first to ophthalmologists are referred to pediatricians to be investigated for other problems associated with these syndromes (47).

Again, it seems likely that there are referrals among hospitalizations, outpatient visits, and nursing home admissions, which are 3 of the sources used by Turabelidze et al. (48) in estimating multiple sclerosis prevalence. Papoz et al. (49) noted that a positive dependency between a list of patients from physicians' practices and a list of patients given prescriptions is “hardly surprising, since the physician is the prescriber.” The tendency for 2 malaria reporting systems to alert each other was observed by Cathcart et al. (50), and Wittes (32) discusses a potential scenario in which some names are actually copied from 1 list to another.

We have demonstrated that, in certain circumstances, an alternative saturated log-linear model might successfully account for referrals. Models 9–11 are based on the assumption of independence of 2 of the sources in the subset of individuals not seen in the third, but on dependence in the subset appearing in the third. Use of these models requires a priori justification of why this might be more plausible for the specific data set than the assumption of no 3-way interaction. More generally, in agreement with Regal and Hook (36) and Cormack (51), we encourage model choice to be guided by an a priori in-depth consideration of the likely relationships among the data sources, guided by discussions with relevant experts, rather than by blind application of some set of statistical criteria. In particular, we urge analysts to explicitly consider whether referrals between data sources are likely. This might be an additional rule to add to the list of recommendations of Regal and Hook (52, 53). If no referrals are present, then standard log-linear methods can be used, although it might be helpful to instead write models directly in terms of meaningful parameters. This facilitates assessment of the plausibility of the estimated parameters, to be used alongside standard model fit statistics to assess model adequacy.

If referrals are believed to be present, then expected cell counts should be formulated in terms of probabilities and proportions, as in Table 3. We have demonstrated that a model parameterized in this way can be fitted using the Bayesian software WinBUGS, but we note that it might also be possible to fit this model using a maximum likelihood approach (36). Our software choice was primarily for pragmatic reasons: WinBUGS automatically provides a credible interval for the population size in addition to a point estimate and makes it simple to enforce constraints such that probability parameters lie between 0 and 1.

For our motivating example, we are not confident that any of the alternative saturated log-linear models (Table 2) is “correct.” For example, the structure assumed by model 9 allows for referrals from both arrest and probation into treatment, but it does not allow for the “standard” interactions that we also expect between these data sources, nor for referrals from arrest into probation. The true expected dependence structure is, unfortunately, too complex for all parameters to be identifiable without additional information. The best advice to those planning a CRC is to avoid this difficult problem by careful selection of data sources, such that complex dependence structures are avoided. If this is impossible, then we encourage the collection of auxiliary information at the point of data matching, such as referral source if known, and/or the specific dates at which individuals appeared in each source. Using this information, it may be possible to quantify the probability that those in overlap sets have been referred, and hence to “remove” likely precaptures prior to analysis.

An alternative solution might be to seek external information to inform the referral parameters. Here, a Bayesian approach could be particularly useful because it offers scope for the incorporation of such external information as informative prior distributions. The external information required need not inform the referral proportions directly; external data can be used to inform a function of parameters within the model (54). However, a great degree of care is needed, because the information must reflect as closely as possible the way in which the specific data were collected, in particular the length of the time window. More generally, a Bayesian framework can be used to incorporate formally other sources of information on prevalence, such as data on drug-related deaths (55). A “multiparameter evidence synthesis” framework (56, 57), in which multiple sources of data are incorporated into a single coherent model, offers potential for formal assessment of the consistency of evidence from different sources. Careful modeling and accounting for known biases of course remain essential.

## APPENDIX 1: STATISTICAL DETAILS

We write λ*_ijk_* for the expectation of each cell count *x_ijk_*. The full log-linear model specification is below, where *I* is the standard indicator function, α is an intercept term, and β_1_ − β_3_ are main effects of inclusion in each of the 3 sources. Source-by-source interaction terms are denoted by δ_12_, δ_13_, and δ_23_, whereas we denote the 3-way interaction term by γ.

$$\eqalign{\log ({\rm \lambda}_{ijk} ) = \ & {\rm \alpha} + {\rm \beta}_1 I(i = 1) + {\rm \beta}_2 I(\,j = 1) \cr & + {\rm \beta}_3 I(k = 1) + {\rm \delta }_{12} I(i = 1)I(\,j = 1) \cr & + {\rm \delta}_{13} I(i = 1)I(k = 1) + {\rm \delta}_{23} I(\,j = 1)I(k = 1) \cr & + {\rm \gamma}I(i = 1)I(\,j = 1)I(k = 1).} $$

This full model, with all 8 possible parameters, implies the following 8 expected cell counts:

**Table:**

S1	S2	S3	${\bf \lambda}_{\boldsymbol{ijk}} $
1	1	1	$\exp ({\rm \alpha} + {\rm \beta}_1 + {\rm \beta}_2 + {\rm \beta}_3 + {\rm \delta}_{12} + {\rm \delta}_{13} + {\rm \delta}_{23} + {\rm \gamma})$
1	1	0	$\exp ({\rm \alpha} + {\rm \beta}_1 + {\rm \beta}_2 + {\rm \delta}_{12} )$
1	0	1	$\exp ({\rm \alpha} + {\rm \beta}_1 + {\rm \beta}_3 + {\rm \delta}_{13} )$
1	0	0	$\exp ({\rm \alpha} + {\rm \beta}_1 )$
0	1	1	$\exp ({\rm \alpha} + {\rm \beta}_2 + {\rm \beta}_3 + {\rm \delta}_{23} )$
0	1	0	$\exp ({\rm \alpha} + {\rm \beta}_2 )$
0	0	1	$\exp ({\rm \alpha} + {\rm \beta}_3 )$
0	0	0	$\exp ({\rm \alpha})$

Now consider, for example, the cross-product of the expected cell counts in the 4 cells representing individuals not seen in source 3.

(1) $$\displaystyle{{{\rm \lambda}_{110} {\rm \lambda}_{000} } \over {{\rm \lambda}_{100} {\rm \lambda}_{010} }} = \displaystyle{{\exp ({\rm \alpha} + {\rm \beta}_1 + {\rm \beta}_2 + {\rm \delta}_{12} )\exp ({\rm \alpha})} \over {\exp ({\rm \alpha} + {\rm \beta}_1 )\exp ({\rm \alpha} + {\rm \beta}_2 )}} = e^{{\rm \delta }_{12} } .$$

As such, if there is no source 1 by source 2 interaction parameter in the model (δ_12_ = 0), then this cross-product must equal 1.

Similarly, because the cross-product in the S3 = 1 cells is

$$\eqalign{&\displaystyle{{{\rm \lambda}_{111} {\rm \lambda}_{001} } \over {{\rm \lambda}_{101} {\rm \lambda}_{011} }} \cr&= \displaystyle{{\exp ({\rm \alpha} + {\rm \beta}_1 + {\rm \beta}_2 + {\rm \beta}_3 + {\rm \delta}_{12} + {\rm \delta}_{13} + {\rm \delta}_{23} + {\rm \gamma}_{123} )\exp ({\rm \alpha} + {\rm \beta}_3 )} \over {\exp ({\rm \alpha} + {\rm \beta}_1 + {\rm \beta}_3 + {\rm \delta}_{13} )\exp ({\rm \alpha} + {\rm \beta}_2 + {\rm \beta}_3 + {\rm \delta}_{23} )}} \cr&= e^{{\rm \delta }_{12} + {\rm \gamma }} ,}$$

then the 3-way cross-product is equal to

(2) $$\displaystyle{{{\rm \lambda}_{111} {\rm \lambda}_{100} {\rm \lambda}_{010} {\rm \lambda}_{001} } \over {{\rm \lambda}_{011} {\rm \lambda}_{000} {\rm \lambda}_{110} {\rm \lambda}_{101} }} = e^{\rm \gamma } .$$

Therefore, if there is no 3-way interaction parameter in the model (γ = 0), then this 3-way cross-product must equal 1 (4).

Using the expected cell counts from Table 3, this 3-way cross product term (equation 2) for our referral scenario 2 is seen to be

$$\displaystyle{{{\rm \lambda}_{111} {\rm \lambda}_{100} {\rm \lambda}_{010} {\rm \lambda}_{001} } \over {{\rm \lambda}_{011} {\rm \lambda}_{000} {\rm \lambda}_{110} {\rm \lambda}_{101} }} = \displaystyle{{\,p_3 + (q_{13} + q_{23} - q_{13} q_{23} )(1 - p_3 )} \over {[\,p_3 + q_{13} (1 - p_3 )][\,p_3 + q_{23} (1 - p_3 )]}}p_3 ,$$

which is equal to 1 only if either *q*_13_ = 0 or *q*_23_ = 0. However, the cross-product in the S3 = 0 cells (equation 1) is equal to 1. This shows that, although an S1xS2xS3 interaction term is needed in the log-linear model to accommodate the referral structure, an S1xS2 interaction term is not. Specifically, the referrals from sources 1 and 2 into source 3 induce a dependency between S1 and S2 in the S3 = 1 cells, but not in the S3 = 0 cells. Model 11 appropriately accommodates this dependence structure.

For our referral scenario 3, we find that the 3-way cross-product (equation 2) is

$$\eqalign{&\displaystyle{{{\rm \lambda}_{111} {\rm \lambda}_{100} {\rm \lambda}_{010} {\rm \lambda}_{001} } \over {{\rm \lambda}_{011} {\rm \lambda}_{000} {\rm \lambda}_{110} {\rm \lambda}_{101} }} \cr&= \displaystyle{{[\,p_1 p_{2|{\rm S1} = 1} + q_{31} (1 - p_1 )p_{2|{\rm S1} = 0} ](1 - p_{2|{\rm S1} = 1} )} \over {\,p_{2|{\rm S1} = 1} [\,p_1 (1 - p_{2|{\rm S1} = 1} ) + q_{31} (1 - p_1 )(1 - p_{2|{\rm S}1 = 0} )]}},}$$

so that a 3-way interaction term must be present in the corresponding log-linear model unless either *q*_13_ = 0 or *p*_2|S1=1_ = *p*_2|S1=0_. Further, consideration of the cross-product in the S2 = 0 cells

$$\displaystyle{{{\rm \lambda}_{101} {\rm \lambda}_{000} } \over {{\rm \lambda}_{001} {\rm \lambda}_{100} }} = \displaystyle{{\,p_1 (1 - p_{2|{\rm S}1 = 1} ) + q_{31} (1 - p_1 )(1 - p_{2|{\rm S}1 = 0} )} \over {(1 - q_{31} )p_1 (1 - p_{2|{\rm S}1 = 1} )}}$$

shows that this is equal to 1 only if *q*_13_ = 0; otherwise, an S1xS3 interaction term must also be present in the corresponding log-linear model. With only 7 observed data points, it is therefore not possible to fit the appropriate log-linear model, which would need to include a 3-way interaction term in addition to all three 2-way interaction terms, and therefore 8 parameters.

## APPENDIX 2: WinBUGS CODE

WinBUGS model code for fitting the correct model for referral scenario 3 (standard S1xS2 and S2xS3 interactions plus referral of a proportion *q*_31_ of individuals from source 3 into source 1).

We assume that the 7 observed cell counts have been formatted into a vector, such that *x*[1] = *x*_111_, *x*[2] = *x*_110_, … , *x*[7] = *x*_001_.

model{

# Split the full multinomial likelihood into two parts

# First part: multinomial likelihood conditional on being observed:

x[1:7] ∼ dmulti(prob.obs[1:7], x.obs)

x.obs <- sum(x[1:7])

for(i in 1:7){

 # Probability of being in the ith cell conditional on being observed:

 prob.obs[i] <- prob[i]/sum(prob[1:7])

}

# Second part: binomial likelihood for being observed

x.obs ∼ dbin(sum.prob, N)

sum.prob <- sum(prob[1:7]) # probability of being observed

xmiss <- N - x.obs       # number of missing individuals

# Specify the 8 cell probabilities directly

# in terms of intuitive parameters:

prob[1] <- (p1*p2S11+ q31*(1-p1)*p2S10)*p3S21

prob[2] <- p1*p2S11*(1-p3S21)

prob[3] <- (p1*(1-p2S11) + q31*(1-p1)*(1-p2S10))*p3S20

prob[4] <- p1*(1-p2S11)*(1-p3S20)

prob[5] <- (1-p1)*(1-q31)*p2S10*p3S21

prob[6] <- (1-p1)*p2S10*(1-p3S21)

prob[7] <- (1-p1)*(1-q31)*(1-p2S10)*p3S20

prob[8] <- (1-p1)*(1-p2S10)*(1-p3S20)

# Vague prior distributions for probabilities:

p1 ∼ dunif(0,1)

p2S10 ∼ dunif(0,1)

p2S11 ∼ dunif(0,1)

p3S20 ∼ dunif(0,1)

p3S21 ∼ dunif(0,1)

# Vague prior for proportion referred from Source 3 into Source 1:

q31 ∼ dunif(0,1)

# Vague prior for total population size:

log.x.obs <- log(x.obs)

log(N) <- logN

logN ∼ dnorm(0, 0.0001)I(log.x.obs,)

}
